# Do Compositions of Lipid Fraction Correspond to Species Differentiation in *Bupleurum* L. (Apiaceae)?

**DOI:** 10.3390/plants9111407

**Published:** 2020-10-22

**Authors:** Zhargal Alexandrovich Tykheev, Oleg Arnoldovich Anenkhonov, Svetlana Vasilievna Zhigzhitzhapova, Vasiliy Vladimirovich Taraskin, Larisa Dorzhievna Radnaeva, Faqi Zhang

**Affiliations:** 1 Baikal Institute of Nature Management, Siberian Branch, Russian Academy of Sciences, 670047 Ulan-Ude, Russia; gagarin199313@gmail.com (Z.A.T.); zhig2@yandex.ru (S.V.Z.); vvtaraskin@mail.ru (V.V.T.); radld@mail.ru (L.D.R.); 2 Laboratory of the Chemistry of Natural Systems, Banzarov Buryat State University, 670000 Ulan-Ude, Russia; 3 Institute of General and Experimental Biology, Russian Academy of Sciences, 670047 Ulan-Ude, Russia; anen@yandex.ru; 4 Key Laboratory of Adaptation and Evolution of Plateau Biota, Northwest Institute of Plateau Biology & Institute of Sanjiangyuan National Park, Chinese Academy of Sciences, Xining 810008, China

**Keywords:** Apiaceae, *Bupleurum*, fatty acids, composition, species differentiation

## Abstract

*Bupleurum* L. has been widely used in various medical systems as an agent with a wide range of activities. The qualitative composition and content of lipid fraction components of the aerial parts of *B. longifolium* and *B. chinense* were elucidated in this work. The available data on the fatty acids (FAs) in *Bupleurum* plants were compiled and compared with species differentiation in the genus. As a result, the content of FAs in the studied *Bupleurum* plant species only partially corresponded to the species differentiation and, in some cases, contradicted it. The prognostic value of *Bupleurum*’s species differentiation for the identification of the potential composition of FAs was insignificant, and it was limited only by particular groups of species. This suggests that a complete phytochemical study of *Bupleurum* species needs to be conducted to determine the composition of FAs and to identify which species have a similar composition.

## 1. Introduction

*Bupleurum* L. is one of the largest genera of the Apiaceae (Umbelliferae) family that includes more than 200 species, most of which are distributed within the Northern Hemisphere (North Africa and Eurasia) [1]. It includes annual and perennial poly- or monocarpic herbaceous plants or, less often, evergreen shrubs with branched caudexes or stem roots [2]. The genus is represented by 36 species in China [3], among which *B. chinense* is one of the most famous and valuable components of Chinese medicine [1,4]. There are 26 species of the *Bupleurum* genus in Russian flora [2]. In traditional medicine existing within the territory of Russia, the aerial parts of plants (*Bupleurum longifolium, B. multinerve, B. scorzonerifolium*, etc.) were used as medicinal agents [5,6,7]. The extensive use of these plants in various medical systems and their treatment efficacy in a range of diseases have led to an increasing number of publications devoted to the study of the chemical composition and pharmacological activity of certain groups of compounds [4]. In general, fatty acids (FAs) have many important biological properties such as energetic, metabolic, and structural activities. Applying unsaturated FAs can alleviate the symptoms of some chronic and degenerative (cardiovascular and inflammatory) diseases [8]. So, the determination of new sources of unsaturated FAs has become one attractive issue in the scientific realm.

There are different systems of species differentiation of *Bupleurum*, and the most developed ones are systematics according to Koso-Polljansky [9] and Wolff [10]. Attempt to establish a correlation between chemical composition and species differentiation of *Bupleurum* have been described in a range of papers. For example, during the identification of *B. chinense*, it has been suggested to use specific markers, such as flavonoids [3], glycosides of phenylpropanoids, lignans, dihydrochalcon, and stilbenoid [11], which characterize exactly this species. The content of different saikosaponins in radices of *B. bicaule*, *B. chinense*, *B. longiradiatum* (=*B. longeradiatum* Turcz.; accepted names according to Catalogue of Life, catalogueoflife.org), *B. falcatum*, *B. scorzonerifolium*, and *B. stenophyllum* used for their identification [12]. The quantitative content of flavonoids has been assumed as possessing the chemotaxonomic significance for *Bupleurum* species: *B. longiradiatum* Turcz. (=*B. longeradiatum* Turcz.), *B. longiradiatum* var. *breviradiatum* (=*B. longeradiatum* Turcz.), *B. sachalinense* Fr. Schmidt (=*B. longeradiatum* Turcz.), *B. euphorbioides*, *B. triradiatum*, *B. triradiatum* var. *alpinum* (=*B. americanum* Coult. & Rose), *B. sibiricum*, *B. nipponicum* Koso-Pol. (=*B. longeradiatum* Turcz.), *B. americanum*, *B. komarovianum*, *B. scorzonerifolium*, *B. scorzonerifolium* var. *stenophyllum* [13].

It is precisely known that the characteristic profiles of FAs encoded by particular genotypes have been widely used as biochemical “fingerprints” for a taxonomy of various plant families. FAs’ profiles and some non-specific and essential FAs have been described as significant genetically determined indicators in the systematic classification and phylogenetic relationships of different plant groups [14]. The composition of FAs can be used as an additional chemometric tool to clarify the taxonomic limits and phylogenetic relationships in the *Bupleurum* genus. From the other side, chemotaxonomic delimitation of species based on the chemical composition of FAs might present the prospect for finding potential sources of FAs. The possibility to characterize the species based on the analysis of the composition of lipid seed biomarkers for *B. croceum*, *B. flavum*, and *B. rotundifolium* from Turkey has been provided earlier [14]. As only seeds have been studied thoroughly, there is a matter of the composition of the entire aerial part and its correlation with species differentiation. This work aimed to understand if there is a correlation between FAs’ composition of the entire aerial part of *Bupleurum* species and its species differentiation for further assessment of the prognostic value of species differentiation to obtain the potential composition of the FAs. For the present, there is information about the FAs’ composition of some plants of the *Bupleurum* genus (Appendix A). However, there are remaining gaps in our knowledge of the FAs’ composition of the aerial parts of some *Bupleurum* species. In part, these gaps for *B. longifolium* and *B. chinense* have been filled in the present study.

## 2. Results and Discussion

The obtained lipid fractions were solid and viscous and had a dark green color and a pleasant smell. The lipid contents were 9.73% and 3.02% in *B. chinense* and *B. longifolium*, respectively. The chemical composition was represented by FAs, sterols, hydrocarbons, and their derivatives (Table 1). A total of 26 different acids were identified in the compositions of the lipid fractions, where the number of carbon atoms was from 10 to 26. The dominant acids of the lipid fractions were 16:0, *cis*18:1n9, and 18:2n9. The total content of these acids in the *B. chinense* sample was 77.44% from the total lipid fraction, and in *B. longifolium*, 58.58%. The content of saturated fatty acids (SFA) in *B. chinense* was 26.05%, in monounsaturated fatty acids (MUFA) was 28.22%, in polyunsaturated fatty acids (PUFA) was 36.85%; in *B. longifolium* SFA—45.91%, MUFA—19.32%, and PUFA—22.35%. Sterols were found only in *B. chinense*. They were represented by stigmasterol (0.62%) and β-sitosterol (0.36%). Alkin 6-tridec-4-yne (0.78%) was also found only in this sample. It should be pointed out that the high content of ketone nonadecanone-10 was identified in both species. This compound was also previously identified in plants of other species: *B. bicaule, B. sibiricum* [15], and *B. scorzonerifolium* [16]. High content of unsaturated FAs, including oleic and linoleic acids, the amount of which was up to 59.12%, provides the pharmacological value of these species.

**Figure 1:**
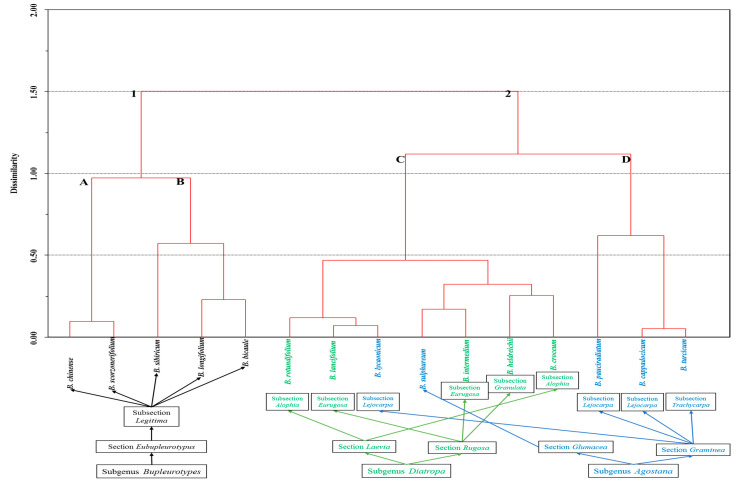
Euclidian dendrogram of the FAs composition from the aerial part of *Bupleurum* L. species based on the results of principal component analysis. Names of infrageneric taxa are given according to Koso-Polljansky [9].

**Figure 2:**
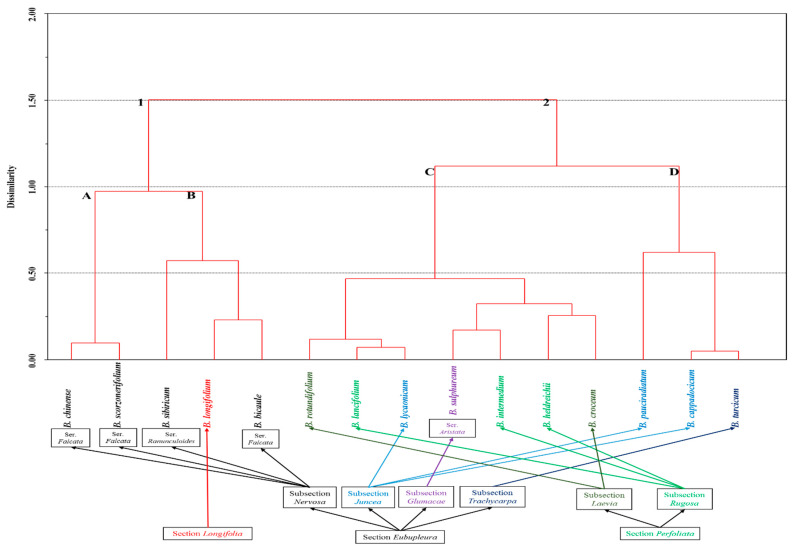
Euclidian dendrogram of the FAs composition from the aerial part of *Bupleurum* L. species based on the results of principal component analysis. Names of infrageneric taxa are given according to Wolff [10].

According to the data obtained and the literature review [14,15,16,17,18,19,20,21,22,23,24], a pool of information on the FAs’ composition in plants of 21 *Bupleurum* species has been gathered (see Appendix A). At the same time, FAs were studied from different parts of the plant: the aerial part, roots, fruits, leaves, seeds, and in different growth stages. As the development of a mature plant from a seed includes many complex chemical transformations that accompany cell division, differentiation of particular organs (roots, stems, leaves, and flowers) and lipids is directly involved in both the formation of membranes and the synthesis of reserve material. As a result, the quantitative composition of FAs formed in the initial period of plant development varies from the composition in the late stages of ontogenesis [25,26,27,28]. Thus, it is necessary to compare the composition of plants on the same developmental stage and from the same morphological parts or the entire aerial part of the plant. For a detailed and optimal discussion of the issue according to the aim of the study, the composition of the entire aerial parts of plants collected in a flowering stage has been chosen. The relative uniformity of the data on FAs in the entire aerial part of *Bupleurum* plants was favorable for this issue. Thus, we conducted a comparative analysis of FAs’ composition of 15 species (see Figure 1 and Figure 2).

SFA with even integers of carbon atoms C12–C26 and unsaturated FAs—16:1n9, cis18:1n9, 18:2n9—were found in all species, but their quantitative content widely varied in different species. These facts confirm the view of T. Ozcan [14] about the possibility to apply FAs’ profiles for chemotaxonomy. The main saturated FA was 16:0. Its content ranged from 10.29% (*B. lancifolium*) to 47.85% (*B. pauciradiatum*). The main monounsaturated FA was cis18:1n9. The lowest and highest concentrations of this compound were found in samples of *B. pauciradiatum* (8.81%) and *B. lancifolium* (69.36%), respectively. The content of polyunsaturated acid 18:2n9 varied from 12.18% (*B. heldreichii*) to 37.12% (*B. chinense*).

The dendrogram obtained based on our results of the FAs composition of *B. chinense* and *B. longifolium* and literature data is presented in Figure 1. According to the concentrations of other acids, *Bupleurum* species were divided, first of all, into perennial and annual plants (Figure 1). Such perennial *Bupleurum* species as *B. bicaule, B. chinense, B. longifolium, B. scorzonerifolium,* and *B. sibiricum* were characterized by the presence of nonanedioic acid and long-chain saturated FAs 22:0, 23:0, 24:0, and 26:0 (Figure 1, group 1). The content of the abovementioned acids in different species varied from 0.30% to 5.61%. Nonanedioic acid (azelaic acid) is an unusual dicarboxylic acid, which was found not only in the aerial parts of perennial *Bupleurum* species but also in the roots of *B. scorzonerifolium, B. chinense* [17], *B. bicaule*, and *B. sibiricum* [15] and in the seeds of *B. falcatum* [18]. Probably, this is revealed as the result of the adaptive mechanisms, taking into account its anti-inflammatory and antibacterial activity. Such saturated 8:0, 11:0, and unsaturated 14:1n9, 15:1n9, 18:3n9 acids were identified in annual species (Figure 1, group 2).

**Figure 3:**
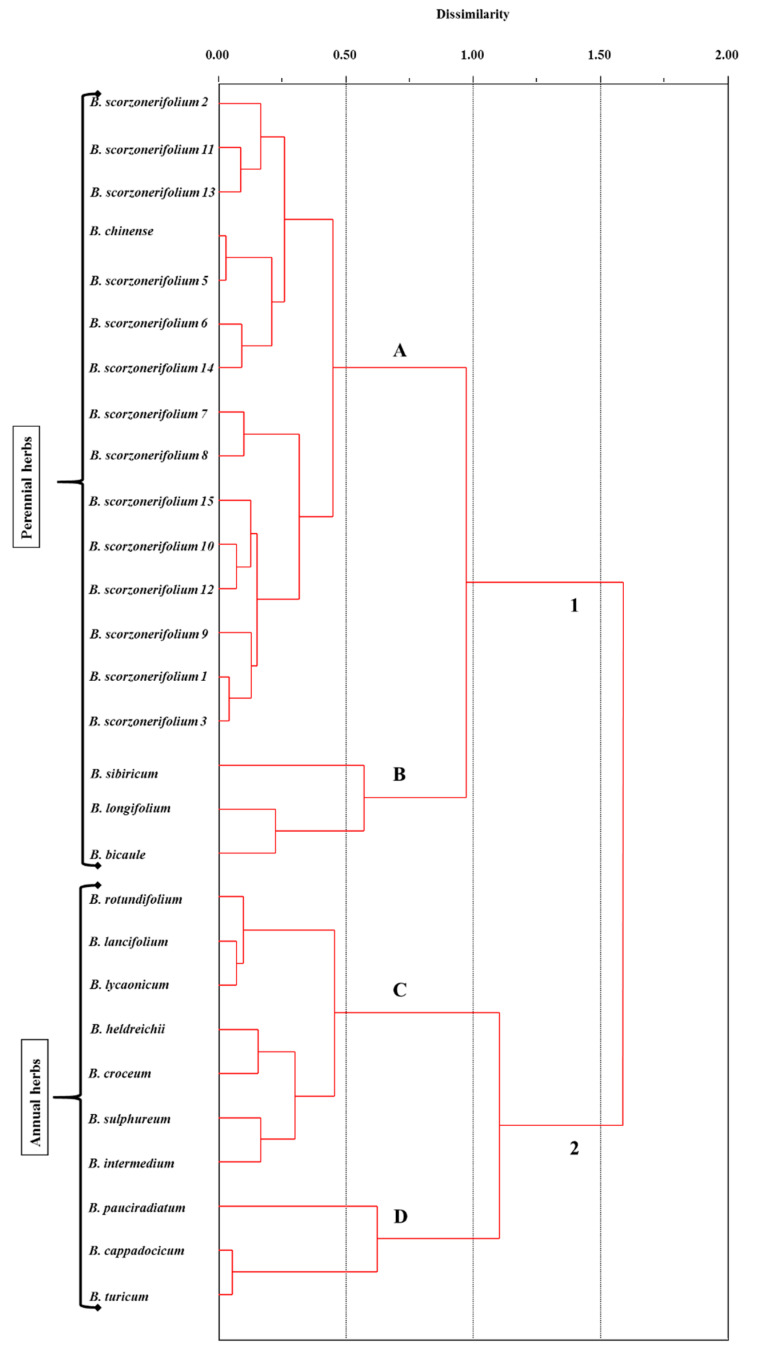
Euclidian dendrogram of the FAs composition from the aerial part of annual and perennial *Bupleurum* L. species displaying *B. scorzonerifolium* from different habitats based on results of principal component analysis.

It appears that the accumulation of long-chain saturated FAs in the lipid composition of perennial species indicates that the adaptation mechanism provides long-term protection from unfavorable factors of the environment, e.g., rapid changes in temperature. In this case, it appears probable that long-chain saturated FAs subsequently were transported to the root system, contributing to the stability under stressful environmental conditions. Apparently, for annual plants, it is not necessary, or energetically unfavorable, to synthesize long-chain FAs. Neves and Watson [29] found that the group, which included only herbs and mainly annual plants (l.c., clades G, H, I in Figure 2 and Figure 3), had longer branches on the phylogenetic tree in comparison with groups including only perennial plants.

Perennial plant species were divided into two subgroups: A and B. Subgroup A included *B. chinense* and *B. scorzonerifolium.* It was characterized by the synthesis of unsaturated *trans*18:1n9 and 16:3n7 FAs in the lipid profile. *B. bicaule, B. longifolium,* and *B. sibiricum* formed subgroup B. This subgroup was associated with the synthesis of unsaturated 16:1n7 acid. Saturated acids were also found in minor amounts in the FAs’ profile of *B. longifolium*—19:0, 21:0, 25:0*—B. bicaul*e, and *B. sibiricum*—28:0. Despite differences in the synthesis of unsaturated acids between subgroups A and B, there is no taxonomical discrepancy revealed between them. Annual species were also divided into two subgroups: C and D. Subgroup C included seven species, and it was characterized by a relatively low content of saturated FAs (12.94–23.28%) and higher content of unsaturated ones (76.72–87.06%). The next subgroup D comprised three species—*B. pauciradiatum*, *B. cappadocicum*, and *B. turcicum—*in which there were relatively high amounts of saturated FAs (32.72–65.20%) and relatively low amounts of unsaturated ones (34.80–67.28%).

It was mentioned that *Bupleurum* is widely distributed throughout the Northern Hemisphere: from the Mediterranean Sea and Macaronesia across Asia to North America, except African endemic species *B. mundii*. Despite the wide distribution of the genus in the world, most of its species have specific geographical areas. *Bupleurum* species grow in various habitats: from sea level to 4900 m a.s.l.; in salinas, lime, granitic, or basalt soils; in arid and mesophytic places; from open areas to dense forests; as weeds and ruderals; and from equatorial to almost arctic forests [2,30]. It is suggested that the final form of the plant, including lipid profile, in our view, is determined by the genotype and epigenetic factors [26]. So, FAs compositions of *B. scorzonerifolium* from different habitats and years of collection [16] did not have an impact on the group classification of perennial and annual plants, but it reflected the rather high intraspecific variability of the FAs composition, which is comparable with the level of variability at the interspecific level in other groups (Figure 1 and Figure 3).

There is no currently accepted species differentiation system of *Bupleurum*. The most developed taxonomy is one according to Koso-Polljansky [9] and Wolff [10]. Analysis of phylogenetic relationships of *Bupleurum* based on the Nuclear Ribosomal DNA Internal Transcribed Spacer Sequence Data did not reveal correlations with the currently known species differentiation of the genus [29]. Comparison of obtained dendrograms with existing systems of species differentiation [9,10] showed that the distribution of samples is the most appropriate to the classification of Koso-Polljansky [9] as supplemented by Linchevskii [31] and Chubarov [32].

The analysis of FAs compositions in the context of a systematic position according to the Koso-Polljansky system [9] determined the following. It showed the formation of cluster 1 for *Bupleurotypes* subgenus, as well as the separation of the subgenera *Diatropa* and *Agostana* into independent subclusters C and D within cluster 2. However, in the latter case, the separation was not completely obvious, as two species of the *Agostana* subgenus (*Bupleurum lycaonicum* and *B. sulphureum*) were similar to representatives of *Diatropa*. The composition of FAs did not have any regularities at the level of sections and especially subsections, e.g., species from the same section did not have similarities with each other but had similar features with species from other sections. Analysis of the similarity in FAs composition at the species level in connection with the systematic position showed the following. According to Chubarov’s [32] sectional positioning of species, there was no FAs similarity between closely related species. Namely, despite the systematic proximity (positioning in one section) and similar ecological-phytocoenotic confinedness [33,34], the composition of FAs of *Bupleurum bicaule* and *B. scorzonerifolium* was significantly different. *B. bicaule* was quite close to *B. longifolium* from another section, which also occupies markedly dissimilar habitats.

*Bupleurum rotundifolium* and *B. lancifolium* had abundantly similar FAs composition, and it corresponded to the results of molecular genetic studies made by Neves and Watson [29]. It was shown that these species are phylogenetically close. Before, they were placed in different sections according to the sectional positioning of species by Koso-Polljansky [9], but they are related to the same section according to Wolff’s system [10] modified by Tutin [35]. Moreover, *B. rotundifolium* and *B. croceum* are located in the same section (*Laevia*), referring to different subclusters within cluster C according to the composition of FAs. Species of the *Leiocarpa* subsection located in different clusters have even more significant differences in FAs composition, e.g., *B. lycaonicum* in cluster C and *B. pauciradiatum* and *B. cappadocicum* in cluster D. Moreover, the latter species according to the FAs composition is similar to *B. turcicum* from another subsection (*Trachycarpa*). Thus, the comparison of the FAs composition in the context of different approaches to species differentiation of *Bupleurum* by different taxonomists showed that differentiation of the FAs composition correlated only at the subgenus level, but even not in all cases.

## 3. Materials and Methods

Various sources were used to gather the data on the FAs composition of plants of *Bupleurum*. First of all, we accumulated our previously published data on the FAs composition of plants of *B. sibiricum* and *B. bicaule* [15] and *B. scorzonerifolium* from different habitats in [16]. Then, we investigated FAs composition from the aerial part of *B. chinense* and *B. longifolium* (see below); these data are reported here for the first time. The data on FAs composition of plants of *B. lycaonicum, B. turcicum*, *B. sulphureum*, *B. heldreichii*, *B. pauciradiatum* were compiled from [20] and data of plants of *B. intermedium*, *B. lancifolium*, *B. croceum*, *B. rotundifolium*, *B. cappadocicum* from [21]. The literature data on FAs in *Bupleurum* plants varied greatly by parts of the plant body, especially data on FAs in seeds, leaves, and roots, which were rather scattered and incomplete. In contrast, the data from the entire aerial part (the aboveground part) of the plant body were fairly uniform. For this reason, we selected the data on FAs in the aerial part of *Bupleurum* plants for further analysis.

*Bupleurum longifolium* L. subsp. *aureum* (Fisch. ex. Hoffm.) Soo was collected in Russia, Republic of Buryatia, Kabanskiy district, the headstream of Bolshoy Mamay river (August 2019, N 51.38 E 104.83, 892 m a.s.l.). *Bupleurum chinense* DC. was collected in China, Qinghai Province, 49 km northeast of Xining (July 2015, N 36.813689, E 102.13302, 2836 m a.s.l.). Samples were collected during the flowering period and air-dried before being ground into a fine powder. Voucher specimens were identified by Dr. Oleg A. Anenkhonov. The voucher specimen of *B. longifolium* was deposited into the Herbarium of IGEB SB RAS (UUH, №№ 018720, 018721, 018722) and *B. chinense* was stored in the local collection of the laboratory for Chemistry of Natural Systems (Baikal Institute of Nature Management SB RAS, TZA-2015-8; Appendix A). Plant names are given according to the Catalogue of Life (http://www.catalogueoflife.org/).

The lipid fraction was isolated by a modified Bligh and Dyer method [36]. A 30~40 g portion of the powdered plant material was extracted with 300 mL of mixture chloroform/methanol (1/2, *v*/*v*) in homogenizer IKA T18 ULTRA-TURRAX T25 (IKA Werke GmbH., Staufen, Germany) for 5 min. Then, the homogenate was filtered, and the plant residues on the filter were again extracted with a mixture of 300 mL of chloroform/methanol (1/2, *v*/*v*) and 80 mL of distilled water. The homogenate was filtered and plant residues on the filter were wetted with a 150 mL mixture of chloroform/methanol (1/2, *v*/*v*). A total of 25 mL of chloroform and 29 mL of distilled water was added to the total extract. Chloroform and water–methanol layers were divided with a separation funnel. The chloroform layer was evaporated under reduced pressure in an IR-50LT rotary evaporator (Labteh, Moscow, Russia) and the extracted lipid fraction was weighed. The lipid fraction content was expressed based on air-dry weight. A total of 200–300 mg of lipid fraction was mixed with 2 mL 2 M hydrochloric acid in methanol in a vial with a screw cap, saponified by heating in a beaker at 90 °C for 2 h. Then, the mixture after cooling was dried under an argon flow to 0.5 mL. One mL of distilled water was added and extracted thrice with 0.5 hexane. The combined hexane fraction was analyzed by gas chromatography–mass spectrometry (GC-MS). To determine the composition of the lipid fraction, GC-MS analysis was performed using an Agilent 6890B gas chromatograph with a 5973N quadrupole mass spectrometer as the detector and an HP-5MS capillary column (30 m × 0.25 mm × 0.2 µm; Hewlett-Packard, Palo Alto, CA, USA). Helium (99.999% purity) was used as the carrier gas at a flow rate of 1.5 mL/min. The oven temperature was programmed as follows: it was kept at a constant temperature of 125 °C for 0.5 min, from 125 to 320 °C (at the rate of 7 °C/min), and kept constant at 320 °C for 0.5 min. The injector and detector temperatures were set to 280 and 250 °C, respectively. The split ratio was adjusted to 40:1. The MS data were acquired in scanning mode at a speed of 2.5 s per scan. The percent composition of the lipid fraction derivatives was calculated from the GC peak areas relative to the total peak area based on the GC-MS analyses of the lipid fraction. Qualitative analysis was based on comparing the retention times and total mass spectra of the corresponding pure compounds using NIST14.L and standard mixtures of Bacterial Acid Methyl Esters (CP Mix, Supelco, Bellefonte, PA, USA) and Fatty Acid Methyl Esters (Supelco, 37 compounds, FAME Mix, 10 mg/mL in CH_2_Cl_2_).

Data were subjected to multivariate statistical analysis using the principal component analysis (PCA). All statistical analyses were conducted using the Sirius software ver. 6.0 (Pattern Recognition Systems, Bergen, Norway) [37] as described in [16].

## 4. Conclusions

A multivariate analysis of the FAs composition of the plants of the *Bupleurum* genus was conducted on the basis of obtained results and literature data. Among others, the qualitative composition and quantitative content of lipid fraction components of the aerial parts of *B. longifolium* and *B. chinense* were reported in this paper for the first time. The chemical composition of the FAs from the aerial parts of the studied *Bupleurum* species is rather diverse. They are distributed unevenly through species and groups of species. However, there was no strong correspondence between the FAs composition and different systems of infrageneric taxonomical division revealed. Instead, the content of FAs in the studied species of the *Bupleurum* genus only partially corresponded to the infrageneric species differentiation and, in some cases, contradicted it. On the other hand, a correlation between the composition of FAs and plant growth form was found, namely the difference in the composition and content of FAs between annual and perennial plants. As a result, the prognostic value of *Bupleurum*’s species differentiation for identification of the potential composition of FAs was more associated not with the plant species but with plant growth form. It was established that perennial species were characterized by the accumulation of long-chain saturated FAs, such as 22:0, 23:0, 24:0, and 26:0, while annuals accumulated a high concentration of saturated 8:0, 11:0, and unsaturated 14:1n9, 15:1n9, 18:3n9 acids. Since plants of the genus *Bupleurum* are valuable medicinal plants, the ability to predict the potential composition of FAs, especially PUFAs, is of great practical importance.

## Figures and Tables

**Table 1:** Chemical composition of the lipid fractions (as methyl esters) from the aerial parts of *Bupleurum chinense* DC. and *Bupleurum longifolium* L. ssp. *aureum* (Fisch. ex. Hoffm.) Soó.

	*B. chinense*	*B. longifolium*
Yield of Lipids, *w*/*w* (%)	9.73	3.02
Compound	Peak Area % (Percentage)	
Saturated fatty acids (SFA)		
10:0		0.12
Nonanedioic acid	0.39	0.36
12:0	0.55	0.90
14:0	1.10	3.31
15:0		1.59
16:0	18.32	20.61
17:0		0.83
18:0	1.98	3.35
19:0		0.18
20:0	0.52	1.19
21:0		0.33
22:0	1.00	2.67
23:0	0.37	2.24
24:0	1.18	3.02
25:0		0.55
26:0	0.64	4.65
∑SFA	26.05	45.91
Monounsaturated fatty acids (MUFA)		
16:1n7		1.85
16:1n9	1.54	1.14
17:1n10		0.19
*cis*18:1n9	25.30	16.14
*trans*18:1n9	0.88	
20:1n1	0.50	
∑MUFA	28.22	19.32
Polyunsaturated fatty acids (PUFA)		
16:3n7	2.53	
18:3n6	0.50	
18:2n9	33.82	21.83
20:3n8		0.52
∑PUFA	36.85	22.35
Others		
6-Tridecene-4-yne	0.78	
10-Nonadecanon	7.12	12.42
Stigmasterol	0.62	
β-Sitosterol	0.36	
∑Others	8.88	12.42
**Total identified**	**100**	**100**

## Appendix A

**Table A1:** Origin and herbarium voucher information of the plant material of *Bupleurum* L. species.

Species	Voucher Number or Mark or Sample No	Country	Locality	Analyzed Parts	Collection Period	Source of Data
*B. longifolium*	UUH018720, UUH018721, UUH018722	Russia	Headstream of Bolshoy Mamay river, Kabanskiy district, Buryatia	Aerial part	August 2019	Present study
*B. chinense*	TZA-2015-8	China	49 km northeast of Xining, Qinghai province	Aerial part	July 2015	Present study
				Roots	July 2015	[17]
	– *		Liaoning province, Shenyang	Roots	–	[19]
*B. bicaule*	–	Russia	Mukhorshibirskiy district, Buryatia	Aerial part and roots	July 2018	[15]
*B. cappadocicum*	HT1008 KNYA	Turkey	Karaman: Karaman–Mut road, Central Anatolia region	Aerial part	May and June 2009	[21]
*B. croceum*	HT1007 KNYA	Turkey	Konya: Altinapa, around Değirmenkoy, Central Anatolia region	Aerial part	May and June 2009	[21]
	–		Native populations distributed in C5, C2, A1, and C2 squares, according to the grid system of Turkey	Fruits	2011–2012	[14]
	TU42168	–	–	Seed and pericarp	–	[22]
*B. falcatum*	–	–	–	Fruits	–	[23]
	–	–	–	Seeds	–	[18]
*B. flavum*	–	Turkey	Native populations distributed in C5, C2, A1, and C2 squares, according to the grid system of Turkey	Fruits	–	[14]
*B. heldreichii*	HT1006 KNYA	Turkey	Central Anatolia region Saracoglu,	Aerial part	May and June 2009	[20]
*B. beldreichii* **	TU41276	–	–	Seed and pericarp	–	[22]
*B. intermedium*	HT1010 KNYA	Turkey	Karaman, Ermenek–Kazanci road, Central Anatolia region	Aerial part	May and June 2009	[21]
*B. lanceolatum*	PA44128	–	–	Seed and pericarp	–	[22]
*B. lancifolium*	HT1009 KNYA	Turkey	Karaman, Ermenek–Kazanci road, Central Anatolia region	Aerial part	May and June 2009	[21]
	B-412	Iran	Khalkhal–Ardabil road, Ardabil province	Leaf and seed	August 2010	[24]
	IS42967TU46887	–	–	Seed and pericarp	–	[22]
*B. longiradiatum*	K044420	–	–	Seed and pericarp	–	[22]
*B. lycaonicum*	HT1004 KNYA	Turkey	Central Anatolia region	Aerial part	May and June 2009	[20]
*B. odontites*	TU46886	–	–	Seed and pericarp	–	[22]
*B. pauciradiatum*	HT1002 KNYA	Turkey	Central Anatolia region	Aerial part	May and June 2009	[20]
*B. rotundifolium*	HT1001 KNYA	Turkey	Konya: in the center of Bozku village, Central Anatolia region	Aerial part	May and June 2009	[21]
	–		Native populations distributed in C5, C2, A1, and C2 squares, according to the grid system of Turkey	Fruits	–	[14]
	–	–	-	Fruits	–	[23]
*B. scorzonerifolium*	TZA-2014-1	Russia	1. Surroundings of the Sotnikovo village, Ivolginsky district, Buryatia	Aerial part and roots	July 2014	[16,17]
	TZA-2015-1		2. Surroundings of the Sotnikovo village, Ivolginsky district, Buryatia	Aerial part	June 2015	[16]
	UUH017463		3. Surroundings of the Sotnikovo village, Ivolginsky district, Buryatia	Aerial part	August 2017	[16]
	TZA-2015-2		5. Surroundings of the Zagustay arbor, Selengisky district, Buryatia	Aerial part and roots	June 2015	[16,17]
	TZA-2015-3		6. Surroundings of the Georgievka village, Khorinsk district, Buryatia	Aerial part and roots	July 2015	[16,17]
	UUH017680		7. Surroundings of the Oninoborsk village, Khorinsk district, Buryatia	Aerial part	August 2016	[16]
	UUH017349		8. Surroundings of the Shiringa village, Eravninsky district, Buryatia	Aerial part	July 2016	[16]
	TZA-2014-3		9. Surroundings of the Nizhniy Tsasuchey village, Ononsky district, Trans-Baikal region	Aerial part	July 2014	[16]
	TZA-2014-4	Mongolia	10. Surroundings of the Bayan Ulaan Uul mountain, Khentii aimag	Aerial part	August 2014	[16]
	TZA-2015-4		11. Surroundings of the Bayan Ulaan Uul mountain, Khentii aimag	Aerial part and roots	August 2015	[16,17]
	TZA-2014-5		12. Surroundings of the Berkh territory, Khentii aimag	Aerial part	August 2014	[16]
	TZA-2015-5		13. Surroundings of the Berkh territory, Khentii aimag	Aerial part and roots	August 2015	[16,17]
	TZA-2014-6		14. In 20 km to the northeast from the Berkh territory, Khentii aimag	Aerial part	July 2014	[16]
	TZA-2014-7		15.Surroundings of the Huh nuur Lake, Khentii aimag	Aerial part	July 2014	[16]
*B. sulphureum*	HT1003 KNYA	Turkey	Central Anatolia region	Aerial part	May and June 2009	[20]
*B. tenue* ***	PA44272	–	–	Seed and pericarp	–	[22]
*B. turcicum*	HT1005 KNYA	Turkey	Central Anatolia region	Aerial part	May and June 2009	[20]

*—Not specified. **—*B. beldreichii* is corrected for *B. heldreichii* ***—is a synonym of *Bupleurum hamiltonii* N.P. Balakr. (according to the Catalogue of Life: http://www.catalogueoflife.org/, and The Plant List: http://www.theplantlist.org/tpl1.1/record/kew-2687216).
